# Dynamic functional connectivity as a neural correlate of fatigue in multiple sclerosis

**DOI:** 10.1016/j.nicl.2020.102556

**Published:** 2021-01-04

**Authors:** Floris B. Tijhuis, Tommy A.A. Broeders, Fernando A.N. Santos, Menno M. Schoonheim, Joep Killestein, Cyra E. Leurs, Quinten van Geest, Martijn D. Steenwijk, Jeroen J.G. Geurts, Hanneke E. Hulst, Linda Douw

**Affiliations:** a Department of Anatomy and Neurosciences, Neuroscience Amsterdam, MS Center Amsterdam, Amsterdam UMC, Vrije Universiteit Amsterdam, Amsterdam, The Netherlands; b Department of Neurology, Neuroscience Amsterdam, MS Center Amsterdam, Amsterdam UMC, Vrije Universiteit Amsterdam, Amsterdam, The Netherlands

**Keywords:** Multiple sclerosis, Fatigue, Resting-state fMRI, Dynamic functional connectivity, Basal ganglia, Default mode network

## Abstract

**Background:**

More than 80% of multiple sclerosis (MS) patients experience symptoms of fatigue. MS-related fatigue is only partly explained by structural (lesions and atrophy) and functional (brain activation and conventional static functional connectivity) brain properties.

**Objectives:**

To investigate the relationship of dynamic functional connectivity (dFC) with fatigue in MS patients and to compare dFC with commonly used clinical and MRI parameters.

**Methods:**

In 35 relapsing-remitting MS patients (age: 42.83 years, female/male: 20/15, disease duration: 11 years) and 19 healthy controls (HCs) (age: 41.38 years, female/male: 11/8), fatigue was measured using the CIS-20r questionnaire at baseline and at 6-month follow-up. All subjects underwent structural and resting-state functional MRI at baseline. Global static functional connectivity (sFC) and dynamic functional connectivity (dFC) were calculated. dFC was assessed using a sliding-window approach by calculating the summed difference (diff) and coefficient of variation (cv) across windows. Moreover, regional connectivity between regions previously associated with fatigue in MS was estimated (i.e. basal ganglia and regions of the Default Mode Network (DMN): medial prefrontal, posterior cingulate and precuneal cortices). Hierarchical regression analyses were performed with forward selection to identify the most important correlates of fatigue at baseline. Results were not corrected for multiple testing due to the exploratory nature of the study.

**Results:**

Patients were more fatigued than HCs at baseline (*p* = 0.001) and follow-up (*p* = 0.002) and fatigue in patients was stable over time (*p* = 0.213). Patients had significantly higher baseline global dFC than HCs, but no difference in basal ganglia-DMN dFC. In the regression model for baseline fatigue in patients, basal ganglia-DMN dFC-cv (standardized β = -0.353) explained 12.5% additional variance on top of EDSS (*p* = 0.032). Post-hoc analysis revealed higher basal ganglia-DMN dFC-cv in non-fatigued patients compared to healthy controls (*p* = 0.013), whereas fatigued patients and healthy controls showed similar basal ganglia-DMN dFC.

**Conclusions:**

Less dynamic connectivity between the basal ganglia and the cortex is associated with greater fatigue in MS patients, independent of disability status. Within patients, lower dynamics of these connections could relate to lower efficiency and increased fatigue. Increased dynamics in non-fatigued patients compared to healthy controls might represent a network organization that protects against fatigue or signal early network dysfunction.

## Highlights

• Fatigue in multiple sclerosis (MS) is highly common but poorly understood.
• Dynamic functional connectivity (dFC) describes variability of brain connectivity.
• Global dFC is higher in MS patients, though this does not relate to fatigue.
• Lower dFC of the basal ganglia and the DMN relates to higher fatigue in patients.

## 1 Introduction

Fatigue is one of the most common symptoms in multiple sclerosis (MS), with up to 83% of patients reporting symptoms of transient or chronic fatigue during the disease course (Manjaly et al., 2019). Fatigue does not have a clear treatment target and is related to a reduced quality of life both through direct effects of fatigue and the strong interrelation between fatigue and comorbidities like depression and impaired cognition (Krupp and Christodoulou, 2001, Comi et al., 2001, Biberacher et al., 2018, Janardhan and Bakshi, 2002). Consequently, gaining an understanding of the neural processes underlying MS-related fatigue is imperative.

MS-related fatigue is thought to have primary causes (i.e. direct effects of MS pathophysiology) and secondary causes (e.g. sleep disorders) (Krupp and Christodoulou, 2001). Various primary (neuro)biological correlates of fatigue in MS have been described in the literature, though the explanatory value is low. Many of these correlates can be fitted into a framework involving physiological alterations in the basal ganglia and frontoparietal regions on various levels of organization. On a molecular level, the release of proinflammatory cytokines is thought to interfere with basal ganglia-cortical loops that affect motivated action, possibly resulting in fatigue (Manjaly et al., 2019, Chaudhuri and Behan, 2000). Studies on the association between MS-related fatigue and structural brain damage (e.g. white matter lesions and atrophy) indicate that regional damage of specific regions such as the thalamus and frontal or parietal cortical regions is a better predictor of for the development of fatigue than global atrophy (Tedeschi et al., 2007, Nourbakhsh et al., 2016, Bakshi et al., 1999, Sepulcre et al., 2009, Pellicano et al., 2010). Functionally, task-based fMRI studies suggest that dysfunction of frontal cortical regions and basal ganglia relates to fatigue in MS patients (Filippi et al., 2002, Roelcke et al., 1997). More recently, resting-state functional connectivity within the Default Mode Network (DMN) and Sensorimotor Network (SMN) has been found to relate to fatigue in MS (Cruz Gómez et al., 2013, Rocca et al., 2018, Høgestøl et al., 2019, Jaeger et al., 2019), though it has been suggested that the DMN may be more strongly involved than the SMN (Bisecco et al., 2018). Moreover, within-basal ganglia functional connectivity and connectivity of the basal ganglia with cortical regions have also been implicated, again mainly involving regions of the Default Mode Network (Rocca et al., 2018, Jaeger et al., 2019, Finke et al., 2015). In addition, reductions in fatigue in response to medication correlate with the modification of these functional basal ganglia-cortical connections, further linking the functional brain network to fatigue in MS patients (Rocca et al., 2018).

Specifically, the subjective experience of fatigue may relate to functional brain network alterations in response to inflammation-induced reductions in network function at rest or during task performance. For instance, abnormal connectivity patterns seen in MS patients during task performance could indicate compensatory recruitment of brain regions not usually involved in execution of this task (Manjaly et al., 2019). From a metacognitive viewpoint, this mismatch between expected and perceived network function could then lead to the subjective experience of fatigue so often seen in MS patients (Leocani et al., 2008). Taking these results together, the analysis of functional network measures could be of importance in better understanding MS-related fatigue.

Traditionally, resting-state functional connectivity is quantified as the average temporal correlation between time series of two brain regions over an entire scanning session. However, recent studies have indicated that functional connectivity in brain networks is far from static and changes dynamically within a single resting-state brain scan (Hutchison et al., 2013). Dynamic functional connectivity (dFC) is a novel functional measure that describes this variability of functional connections between brain regions over time and is related to behavioral and cognitive state in healthy subjects (Cohen, 2018, Lurie et al., 2020, Braun et al., 2015, Douw et al., 2016, Fong et al., 2019). In some contexts (e.g. cognitive flexibility during a test of cognitive inhibition) higher task-state dFC and lower resting-state dFC in specific resting-state networks relate to better performance (Douw et al., 2016). In contrast, during other tasks (e.g. focused attention) lower dFC overall is associated with better performance (Fong et al., 2019). These opposing results highlight the context-specific relevance of dFC, depending on the *state* (e.g. rest, motor task, or cognitive task) and *location*, i.e. brain regions or networks considered. Additionally, perturbations in dFC are found in various neurological diseases, suggesting that specific pathophysiological processes may be reflected in changes in the variability of the brain network (Lurie et al., 2020). For MS specifically, dFC has been associated with white matter lesion load and cognitive (dys)function (Huang et al., 2019, Eijlers et al., 2019, van Geest et al., 2018, van Geest et al., 2018, d’Ambrosio et al., 2020). Considering the framework described earlier, in which subjective experience of fatigue in MS is thought to be tied to network function, (disturbed) dFC may relate to fatigue in these patients as well.

The aim of this exploratory study is to investigate whether the novel measures of resting-state dFC can provide additional insight into the neural correlates of fatigue in MS. We use measures of global and regional dFC to study the relationship with fatigue, in order to assess the descriptive value of network dynamics for fatigue. We expected to find differences in dFC in MS patients compared to healthy controls, reflecting widespread inflammation-moderated network alterations. Moreover, we hypothesized that dFC between the basal ganglia and cortical regions would correlate with fatigue in MS patients as functional loops involving these regions have been found to relate to MS-related fatigue previously.

## 2 Methods and materials

### 2.1 Subjects

The study sample consisted of MS patients (n = 35) and healthy controls (HCs; n = 19) matched for age, sex, and education (using the Verhage scale) (van Geest et al., 2018). Inclusion criteria for all subjects were: (1) compliance with all safety indications for MRI; (2) age between 18 and 65 years; (3) no history or presence of psychiatric or neurological disease (apart from MS for the patient group); (4) no history or presence of alcohol or drug abuse. The patient group met the additional inclusion criterion of a diagnosis of relapsing-remitting MS. Patients were excluded from the study if they experienced a relapse or underwent steroid treatment in the 4 weeks prior to examination. Moreover, subjects would be excluded from analysis if they exhibited excessive frame-to-frame head motion during resting-state fMRI (head displacement of >0.5 mm for 20% of frame-to-frame transitions) in order to reduce potential confounding effects on measures of dFC. This was not the case for any subject. The patient group consisted of 18 patients that recently switched from first-line treatment to fingolimod treatment and 17 patients that continued first-line treatment (glatiramer acetate, interferon beta, teriflunomide, or dimethyl fumarate), matched for disease duration. The cohort used for analysis here is part of a longitudinal study into the clinical effects of fingolimod on MS patients, which was approved by the local institutional ethics review board. There has been one prior publication on this dataset with a focus on correlates of cognition (van Geest et al., 2018). All subjects provided written informed consent.

### 2.2 Clinical measures and fatigue

Clinical measures were assessed at baseline (T0) and at follow-up 6 months later (T1). The patient group underwent physical examination, yielding scores on the Expanded Disability Status Scale (EDSS) (Kurtzke, 1983). Additionally, we administered several questionnaires in both groups. Self-reported fatigue was assessed using the revised Checklist of Individual Strength (CIS-20r), which has been validated for use in MS patients (Vercoulen et al., 1994, Elbers et al., 2012). In the CIS-20r questionnaire, subjects were asked to rate their agreement with 20 statements on self-experienced fatigue in the past two weeks on a scale from 1 to 7. This questionnaire resulted in fatigue scores on the following four subdomains: subjective complaints (8 questions), motivation (4 questions), physical activity (3 questions), and concentration (5 questions). A total fatigue score was obtained by summing the subdomain scores. The Hospital Anxiety and Depression Scale (HADS) was used to measure self-reported anxiety and depression (Zigmond and Snaith, 1983).

### 2.3 Structural and functional MRI

At baseline, MRI data of each subject were acquired using a 3T magnet whole-body MRI system (GE Signa-HDxt, Milwaukee, WI, USA) with a 32-channel phased-array head coil. The protocol contained a three-dimensional T1-weighted (3DT1) sequence for brain volume measurements (TR: 8.22 ms; TE: 3.22 ms; TI: 450 ms; flip angle 12°; 1.0 mm sagittal slices; 0.94 mm in-plane resolution) and a fluid-attenuated inversion recovery (FLAIR) sequence for detection of white matter lesions and their quantification (TR: 8000 ms; TE: 128 ms; TI: 2343 ms; 1.2 mm sagittal slices; 0.98 mm in-plane resolution). In addition, an eyes-closed resting-state fMRI was performed, yielding 202 volumes of echo planar images (TR: 2200 ms; TE: 35 ms; flip angle 80°; 3.0 mm axial slices; 3.3 mm in-plane resolution). All structural and functional MRI data were processed with the FMRIB Software Library v6.0 (FSL, fmrib.ox.ac.uk/fsl). For MS patients, white matter lesions on FLAIR images were segmented and LEAP was used to fill them on T1-weighted images automatically (Chard et al., 2010). Subsequently, total gray matter and white matter volumes were measured with SIENAX (Smith et al., 2002). FIRST segmentation was used to calculate subcortical gray matter volumes (Patenaude et al., 2011). Then, cortical gray matter volume was estimated by subtracting the FIRST subcortical segmentation from the total gray matter volume obtained by the SIENAX segmentation. The SIENAX v-scaling factor was used to normalize all volumetric measurements for head size.

Functional MRI data were preprocessed with FSL MELODIC during which: (1) the first five volumes were discarded; (2) MCFLIRT motion correction was applied; (3) spatial smoothing with a Gaussian filter (full-width-at-half-maximum: 6.0 mm) was applied. Prior studies have described the large effects that motion artifacts can have on the detection of dFC (Cohen, 2018). Therefore, additional motion artifacts were removed from functional data using ICA-AROMA, a tool that uses independent component analysis to remove motion-induced noise from fMRI data (Pruim et al., 2015). Afterwards, residual signal from white matter and CSF was regressed out and high-pass filtering was applied with a cutoff frequency of 0.01 Hz. To define nodes, the Brainnetome atlas was used for cortical regions and FIRST regions for the deep gray matter (Fan et al., 2016). The cortical Brainnetome atlas was inversely registered to structural subject space and multiplied with the SIENAX gray matter mask, providing 210 cortical gray matter regions for each individual subject. After this, the FIRST-based parcellation of subcortical gray matter structures was added to the atlas, together providing a total of 224 regions. Subsequently, this structural atlas was co-registered to functional space for each subject, using inverted boundary-based registration parameters. Here, the atlas was multiplied with an fMRI mask based on the field of view, together with the exclusion of voxels that had a signal intensity in the lowest 25% of the intensity distribution robust range (e.g. regions too severely distorted by artifacts). After multiplying with this mask, individual regions were assessed whether sufficient coverage remained. Finally, the average signal intensity was calculated for each volume, forming the time series for each anatomical region of the final atlas.

### 2.4 Connectivity measures

**Fig. 1:**
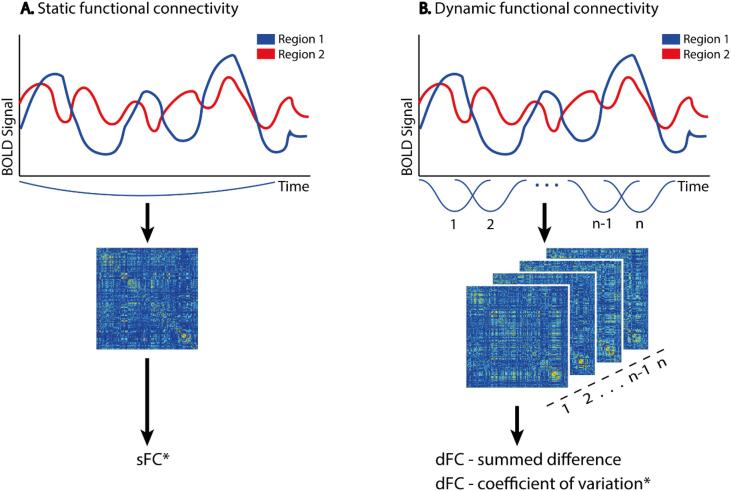
Calculations of functional connectivity. For each subject, static functional connectivity during the entire time series was calculated. Moreover, the time series was divided into overlapping windows (length of 27 volumes, 59.4 s; shift of 5 volumes, 11 s). From these windows, global dynamic functional connectivity was calculated using the summed difference method and the coefficient of variation. *: absolutized connectivity matrices were used.

Connectivity measures were calculated in Matlab 2018a (Natick, MA, USA). Static functional connectivity was assessed by calculating the average absolute Pearson correlation coefficient during the entire time series for each pair of regions, resulting in a 224 × 224 undirected connectivity matrix for each subject (Fig. 1A). Dynamic functional connectivity was calculated by using a sliding-window approach (Fig. 1B): the entire time series of the functional data was divided into overlapping windows with a pre-specified length (27 volumes; 59.4 s) and shift from one window to the next with a gap of 5 volumes (11 s) between windows. It has been established prior that this approximate window length is appropriate for measuring fluctuations in resting-state functional connectivity (Preti et al., 2017). In order to reduce the effects of high-frequency noise on dFC estimates, this square window was convolved using a Gaussian kernel (standard deviation: 9 TR), creating a tapered window (Huang et al., 2019). Subsequently, we calculated the weighted Pearson correlation coefficient between all pairs of brain regions using the weights of the tapered window. Subsequently, we estimated dFC using two different methods to scrutinize two distinct aspects of variability in functional connectivity: (1) the summed difference (diff) and (2) the coefficient of variation (cv). In order to calculate the summed difference, the absolute difference in connectivity from one window to the next was calculated and summed for each individual cell in the matrix. The coefficient of variation over all windows (using absolutized connectivity matrices) was calculated for each individual connection with the following formula, where *i* indicates the connection between two distinct brain regions: $\mathrm{cv}\left(i\right)=\frac{\sigma_{i}}{\mu_{i}}$, in which µ indicates the average connection strength between the regions and σ the standard deviation of the connection strength over all windows. Whereas the summed difference focuses solely on connectivity changes from one window to the next, the coefficient of variation describes changes in connectivity in the context of all windows and overall static connectivity.

Both approaches yielded symmetrical 224 × 224 matrices for each subject, in which each value indicated how much connectivity between a single pair of brain regions changed over time. Within each of the three final connectivity matrices, all cells were averaged to obtain a value for global sFC, global summed difference dFC, and global coefficient of variation dFC for each subject.

### 2.5 Regional functional connectivity measures

**Fig. 2:**
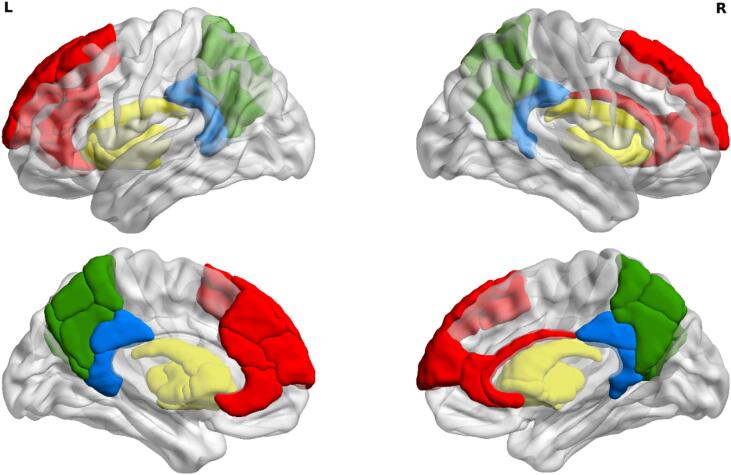
Brain regions used for analysis of basal ganglia-DMN connectivity. The top panel presents a lateral view of the left (L) and right (R) hemisphere and the bottom panel a medial view. Regions included for analysis were the medial prefrontal cortex (red), posterior cingulate cortex (blue), precuneus (green), and basal ganglia (yellow). Areas were visualized using BrainNet Viewer (Xia et al., 2013). (For interpretation of the references to color in this figure legend, the reader is referred to the web version of this article.)

To study regional connectivity patterns, functional connectivity between anatomical regions that have been consistently found to be associated with fatigue was calculated as well (i.e. the basal ganglia and cortical regions in the DMN). Regions that we selected were the bilateral basal ganglia (caudate nucleus, putamen, globus pallidus) and the bilateral medial prefrontal cortex, posterior cingulate cortex, and precuneus (Fig. 2) (Høgestøl et al., 2019, Bisecco et al., 2018, Finke et al., 2015). Interregional functional connectivity between all these selected regions was extracted from the final 224 × 224 connectivity matrices and averaged for each of the connectivity measures calculated (sFC, dFC summed difference, dFC coefficient of variation), yielding one value for basal ganglia-DMN connectivity. In order to correct for differences in the strength of these connections due to intersubject global connectivity differences, basal ganglia-DMN connectivity values were divided by the global connectivity value for each subject.

### 2.6 Null model for dFC

Methodologically, the sliding-window approach might lead to a classification of spurious fluctuations in functional connectivity as true dynamic connectivity due to increased sampling variability that arises from decreasing window size (Lurie et al., 2020, Hindriks et al., 2016). Hence, the need for appropriate statistical testing for a sliding-window approach has been emphasized. In order to test for the presence of nonspurious dFC, we created 100 randomized time-series using phase randomization on the discrete Fourier transform of the time series, in which sFC and autocorrelation remain preserved (described in detail elsewhere: Hindriks et al., 2016, Prichard and Theiler, 1994). Taking the discrete inverse Fourier transform afterwards yielded the randomized copy of the time series. This procedure yielded 100 randomized sets of time series per subject. After obtaining the randomized surrogate time series, average global and basal ganglia-DMN dFC (without normalization) were calculated over all randomizations for each subject, calculating both the summed difference and the coefficient of variation. Using paired sample *t*-tests, differences between mean dFC in real and surrogate data were calculated for each measure of dFC. For each of the dynamic measures calculated, dFC obtained from real data was significantly higher than the dFC obtained from surrogate data, indicating the presence of nonspurious dFC in our data (Table S1).

### 2.7 Statistical analyses

For statistical analyses, IBM SPSS version 22 was used (Armonk, NY, USA). Histogram inspection and Kolmogorov-Smirnov tests were used to assess normality of variables, and nonparametric tests were used in cases of non-normality. Independent two-sample *t*-tests and Mann-Whitney U-tests were carried out accordingly to investigate cross-sectional differences between HCs and MS patients for demographics, anxiety, depression, and fatigue. Furthermore, cross-sectional tests were performed in order to assess differences in structural volumetric measurements, dFC, and sFC between HC and MS patients at baseline. For both groups, longitudinal tests (paired sample *t*-test or Wilcoxon signed-rank test) were performed to investigate progression on fatigue and clinical measures from baseline to 6-month follow-up.

Next, we used a hierarchical forward regression model in order to determine the predictive value of dFC for total fatigue score of MS patients and HCs at baseline in combination with several other predictors. The first block in this regression model included the following predictors: age, sex, educational level, medication used (only in MS), disease duration (only in MS), and EDSS at baseline (only in MS). The second block consisted of baseline normalized gray matter volume, white matter volume, and log-normalized lesion volume (only in MS). The third and final block included global and basal ganglia-DMN dFC measured with the summed difference and coefficient of variation at baseline. None of these predictors showed issues of collinearity prior to performing regression analysis.

Based on the outcome of the regression model, we performed several post-hoc tests. Firstly, we calculated pairwise correlations between predictors that ended up as significant predictors in the regression model and fatigue scores (total score and subscales) for MS patients using Spearman’s rank correlation. Furthermore, we tested for the clinical relevance of the predictors of the regression model. To do so, the MS sample was divided into a non-fatigued and a fatigued group at both time points based on the CIS-20r cutoff value of 76 for severe fatigue, after which we tested group differences in the predictor values between healthy controls, non-fatigued patients, and fatigued patients (Bültmann et al., 2000). P-values lower than 0.05 were considered statistically significant. Due to the exploratory nature of the study and the strong interrelations between variables explored, results were not corrected for multiple testing.

## 3 Results

### 3.1 Demographics and sample description

At baseline, the sample consisted of 35 MS patients and 19 healthy controls. At 6-month follow-up, one patient dropped-out as a result of discontinued interest in the study and another due to excessive fatigue. Both drop-outs were using first-line medication. No differences were found between MS patients and HCs for age, sex, and educational level (Table 1). The two medication groups (fingolimod and first-line disease-modifying treatment) showed no differences in mean disease duration, baseline and follow-up EDSS, and baseline and follow-up fatigue (Table S2).

**Table 1:** Demographics at baseline.

	MS (*n* = 35)	HC (*n* = 19)	Test statistic	*p*
Age	42.83 (10.39)	41.38 (13.27)	t(30.2) = −0.414	0.682
Sex (female/male)	20/15	11/8	$\chi^{2}$(1) = 0.003	0.957
Educational level^a^	6.00 (5.00–7.00)	6.00 (5.00–7.00)	$\chi^{2}$(2) = 4.586	0.101
Disease duration	10.97 (7.00)	–	–	–

Data shown here are mean (standard deviation). HC = healthy controls; MS = multiple sclerosis.

a Data are median (range) and were measured using the Verhage education scale.

At baseline, no differences were found for anxiety and depression between MS patients and HCs (Table 2). At follow-up, no differences in anxiety were observed between patients and HCs, but depression levels were higher at follow-up in the MS patients compared to HCs (*p* = 0.006). No longitudinal changes in EDSS and HADS were observed within each group.

**Table 2:** Clinical measures and questionnaires at baseline and 6-month follow-up.

		Group (n(Baseline), n(Follow-up))		Test statistic	*p*
		MS (*n* = 35, *n* = 33)	HC (*n* = 19, *n* = 19)		
EDSS	Baseline	3.00 (1.00–6.00)	–	–	–
	Follow-up	3.00 (1.50–7.00)^a^	–	–	–
	Test statistic	Z = -0.804	–		
	*p*	0.422	–		

HADS-A	Baseline	5.50 (0.00–12.00)^b^	4.00 (1.00–13.00)	U = 247	0.156
	Follow-up	6.00 (0.00–17.00)^a^	4.00 (1.00–13.00)	U = 252	0.306
	Test statistic	Z = -0.138	Z = -0.884		
	*p*	0.890	0.376		

HADS-D	Baseline	3.00 (0.00–14.00)^b^	1.00 (0.00–6.00)	U = 230	0.080
	Follow-up	3.00 (0.00–9.00)^a^	1.00 (0.00–12.00)	U = 165.5	0.006*
	Test statistic	Z = -0.420	Z = -0.354		
	*p*	0.674	0.723		

CIS-20r Total	Baseline	74.36 (29.33)^c^	46.72 (17.06)^d^	U = 134.5	0.001*
	Follow-up	69.91 (27.01)^a^	45.11 (19.84)	U = 147	0.002*
	Test statistic	Z = -1.245	Z = -0.104		
	*p*	0.213	0.918		

Data shown here are median (range). HC = healthy controls; MS = multiple sclerosis. EDSS = Expanded Disability Status Scale; HADS = Hospital Anxiety and Depression Scale; A = Anxiety; D = Depression. CIS-20r = Revised Checklist of Individual Strength.

a n = 32.

b n = 34.

c n = 33.

d n = 18.

### 3.2 Fatigue

Fatigue scores are shown in Table 2, Table 3. At baseline, MS patients reported higher overall fatigue, measured with the CIS-20r total score (*p* = 0.001), compared to HCs. Significantly higher scores were seen on all fatigue subscales: subjective complaints (*p* = 0.002), motivation (*p* = 0.010), physical activity (*p* = 0.001), and concentration (*p* = 0.012). At baseline, 54.5% of MS patients had a total fatigue score of 76 or higher, the clinical cutoff value for severe fatigue (Bültmann et al., 2000). At 6-month follow-up, MS patients still exhibited greater total fatigue than HCs (*p* = 0.002). At follow-up, the percentage of MS patients with a fatigue score higher than the cutoff value for severe fatigue was 50.0%. No changes in fatigue scores between baseline and 6-month follow-up were observed for MS patients and HCs.

**Table 3:** Cross-sectional fatigue measures of CIS-20r subscales at baseline.

	MS (*n* = 35)	HC (*n* = 19)	Test statistic	*p*
CIS-20r/S	34.12 (15.28)^a^	20.79 (8.87)	U = 151.5	0.002*
CIS-20r/M	12.58 (6.55)^a^	7.89 (3.60)	U = 178	0.010*
CIS-20r/P	10.00 (5.22)^a^	5.26 (2.40)	U = 137	0.001*
CIS-20r/C	17.69 (7.37)^a^	12.39 (6.96)^b^	U = 170.5	0.012*

Data shown here are mean (standard deviation). HC = healthy controls; MS = multiple sclerosis. CIS-20r = Revised Checklist of Individual Strength; S = subjective complaints; M = motivation; P = physical activity; C = concentration.

a n = 33.

b n = 18.

### 3.3 MRI measures

No differences in white matter volume were observed between patients and HCs (Table 4). However, MS patients had lower cortical gray matter volume (*p* = 0.002), as well as lower subcortical gray matter volume (p < 0.001). HCs and MS patients showed no difference in global sFC at baseline. Global dFC in MS patients was higher using both the summed difference method (*p* = 0.025) and coefficient of variation (*p* = 0.012). None of the basal ganglia-DMN connectivity measures were different between MS patients and HCs.

**Table 4:** MRI measures at baseline.

	MS (*n* = 35)	HC (*n* = 19)	Test statistic	*p*
NWMV (ml)	679.16 (44.46)	697.24 (39.08)	t(52) = 1.487	0.143
NCGMV (ml)	744.57 (78.30)	810.51 (56.32)	t(52) = 3.238	0.002*
NSGMV (ml)	56.95 (8.50)	64.95 (4.49)	t(52) = 4.524	<0.001*
NLV (ml)	24.73 (17.28)	–	–	–

Global sFC	0.24 (0.06)	0.26 (0.06)	U = 232	0.069
Global dFC-diff	3.38 (0.25)	3.21 (0.28)	t(52) = −2.316	0.025*
Global dFC-cv	0.60 (0.04)	0.58 (0.04)	U = 194	0.012*

BD sFC	1.32 (0.22)	1.37 (0.24)	t(52) = 0.822	0.415
BD dFC-diff	0.95 (0.06)	0.93 (0.05)	t(52) = −1.325	0.191
BD dFC-cv	0.91 (0.06)	0.87 (0.08)	t(52) = −1.810	0.076

Data shown here are mean (standard deviation). HC = healthy controls; MS = multiple sclerosis. NWMV = normalized white matter volume; NCGMV = normalized cortical gray matter volume; NSGMV = normalized subcortical gray matter volume; NLV = normalized lesion volume; sFC = static functional connectivity; dFC-diff = dynamic functional connectivity – summed difference method; dFC-cv = dynamic functional connectivity – coefficient of variation; BD = basal ganglia-DMN.

### 3.4 (Neural) correlates of fatigue

Table 5 shows the results of the forward regression analysis for HCs and MS patients for total fatigue at baseline and at 6-month follow-up. For baseline fatigue in MS patients, the final regression model explained 26.0% of variance (F(2,30) = 5.269, *p* = 0.011) with EDSS (standardized β = 0.380, *p* = 0.022) and basal ganglia-DMN dFC coefficient of variation (standardized β = −0.353, *p* = 0.032) selected as predictors. Adding basal ganglia-DMN dFC assessed with the coefficient of variation to the model on top of EDSS increased the variance explained by the model with 12.5% (F(1,30) = 5.053, *p* = 0.032). For baseline total fatigue in HCs, the final model explained 22.4% of the variance in fatigue (F(2,15) = 4.625, *p* = 0.047), with the only predictor for higher fatigue being male sex (standardized β = 0.474, *p* = 0.047).

**Table 5:** Outcomes of final hierarchical regression model for baseline and follow up total CIS-20r score.

Group	Dependent variable	Predictors	R^2^	Adjusted R^2^	Standardized β	Test statistic	*p*
MS	T0 CIS-20r		0.260	0.211		F(2,30) = 5.269	0.011*
		*T0 EDSS*			0.380	t = 2.418	0.022*
		*BD dFC-cv*			−0.353	t = -2.248	0.032*
HC	T0 CIS-20r		0.224	0.176		F(1,16) = 4.625	0.047*
		*Sex*			0.474	t = 2.151	0.047*

CIS-20r = Revised Checklist of Individual Strength; T0 = Baseline; EDSS = Expanded Disability Status Scale; BC = basal ganglia-DMN; dFC-diff = dynamic functional connectivity – summed difference method; dFC-cv dynamic functional connectivity – coefficient of variation.

Fig. S1 shows the post-hoc correlation analysis of fatigue predictors (EDSS and basal ganglia-DMN dFC-cv) with fatigue subscales and total scores for MS patients. Basal ganglia-DMN dFC-cv showed a negative correlation with three out of five fatigue scores at baseline and did not correlate with disability.

### 3.5 Post-hoc: predictor values in fatigued and non-fatigued patients

In Table 6, the significant predictors of the regression model are compared between non-fatigued and fatigued MS patients based on the clinical cutoff value (CIS-20r ≥ 76), with healthy controls included as a reference point. There was a significant difference in basal ganglia-DMN dFC-cv between the three groups (*p* = 0.038). Post-hoc pairwise comparisons showed a significant difference between healthy controls and non-fatigued patients (*p* = 0.013), but not between healthy controls and fatigued patients (*p* = 0.57) or between non-fatigued patients and fatigued patients (*p* = 0.056). EDSS was not different between non-fatigued and fatigued MS patients.

**Table 6:** Values of baseline regression model predictors in non-fatigued and fatigued MS patients.

	HC (*n* = 19)	NF (*n* = 15)	F (*n* = 18)	Test statistic	*p*
BD dFC-cv^a^	0.91 (0.06)	0.93 (0.05)	0.89 (0.06)	H(2) = 6.54	0.038*
T0 EDSS^b^	–	2.50 (1.00–6.00)	3.25 (1.50–6.00)	U = 89	0.100

NF = non-fatigued; F = fatigued; BD = basal ganglia-DMN; dFC-diff = dynamic functional connectivity – summed difference method; dFC-cv = dynamic functional connectivity – coefficient of variation. sFC = static functional connectivity.

a Data are mean (standard deviation).

b Data are median (range).

## 4 Discussion

In this study, dFC was measured in MS patients and HCs in order to explore its relationship with fatigue. Global dFC was higher in MS patients than in HCs, though this measure did not relate to fatigue. Yet, basal ganglia-DMN dFC had additional explanatory value for total fatigue at baseline. In patients, lower basal ganglia-DMN dFC correlated with higher fatigue scores at baseline and did not correlate with disability. Lastly, non-fatigued MS patients showed higher basal ganglia-DMN dFC than healthy controls, whereas fatigued MS patients did not differ from either group.

In line with previous literature describing functional connectivity alterations in MS patients overall, patients in this study showed greater global dFC compared to HCs. Previous research in MS patients has shown both locally and globally altered patterns of resting-state sFC compared to healthy controls (Bassi et al., 2007). Moreover, local differences in dFC have been found in MS patients compared to HCs, correlating with white matter lesion load (Huang et al., 2019). These alterations in sFC and dFC indicate widespread network changes in the brains of MS patients that co-occur with the development of pathology and structural damage. The exact nature of these network modifications are poorly understood and may represent a combination of inflammation-induced network disturbances and active functional network adaptations in response to these disturbances (Bassi et al., 2007). Though global dFC was higher in MS patients than in healthy controls, no relationship between global dFC and fatigue was found. An explanation for this could be the high context-specificity of dFC that has been established earlier. The utility of dFC and its relationship to symptoms has been found to be highly dependent on regions and functional networks considered (Eijlers et al., 2019, van Geest et al., 2018, van Geest et al., 2018, d’Ambrosio et al., 2020). Moreover, fatigue in MS patients may be a symptom that is better explained by disturbances in specific structural and functional networks (Manjaly et al., 2019). It is possible that global dFC (a rather general measure), though increased, does not have a specific relationship to fatigue in MS patients.

Interestingly, region-specific dFC (i.e. basal ganglia, posterior cingulate cortex, medial prefrontal cortex, and precuneus) turned out to be a significant predictor for total fatigue independently of physical disability. More specifically, within the patient group, patients with lower basal ganglia-DMN dynamic connectivity displayed more severe fatigue. These findings can be placed into context by using healthy controls as a reference point and by using clinical cutoff scores for MS patients. Here, patients without severe fatigue showed higher basal ganglia-DMN dFC compared to healthy controls, whereas basal ganglia-DMN dFC between healthy controls and severely fatigued patients was not different. It is hypothesized that the optimal brain network depends on the right balance between stable segregation and dynamic integration (Sporns, 2002). Thus, one may speculate that functional dynamics are important for the efficiency of the functional loops that connect the basal ganglia and cortical regions of the DMN. For this subset of connections, an increase in network dynamics in patients compared to controls could correspond to increased network efficiency. This increase in efficiency may compensate for brain-wide network impairments due to MS pathophysiology, and protect against severe fatigue. However, the exact relationship between dFC and fatigue (i.e. the role of network dynamics of these connections in relation to network efficiency) may differ between patients and controls. Alternatively, this increase could signal early network dysfunction which may actually lead to fatigue in the long run. These alternative conclusions cannot be ruled out based on our data, so interpretation of these results should be done with caution.

Following the framework described above, the absence of these increased dynamics in the patient population could correspond to an inability to compensate for widespread network impairments. This would then relate to a higher degree of fatigue, as was found in the regression analysis. The absence of a difference in basal ganglia-DMN dFC between non-fatigued and fatigued MS patients could be the result of decreased power due to the binarization of fatigue using the clinical cutoff. Our findings also further corroborate the role of the basal ganglia in fatigue, as has been found in prior research into structural and functional correlates of fatigue in MS as well as other neurological diseases (e.g. Parkinson’s disease) (Chaudhuri and Behan, 2000). The reason for this could be that the basal ganglia and their connections to cortical regions comprise functional loops important for goal-based behavior and motivated action, which might be impaired in fatigued patients across diseases (Manjaly et al., 2019). As such, the relationship of dFC of these connections with more specialized motivational aspects of fatigue is a potentially interesting topic for future studies.

Besides a relationship between fatigue and dFC in MS patients, the present study also established a connection between fatigue and EDSS. This association between disability and fatigue in MS patients has been found previously and may be stronger with physical fatigue than with other types of fatigue (Biberacher et al., 2018). The current study did not identify global structural MRI measures such as white matter volume, gray matter volume, and white matter lesion volume as predictors for fatigue in MS patients. The relationship between fatigue and structural MRI measures has been highly debated in prior research. Although some studies did find global structural damage (lower gray and white matter volume and higher lesion volume) to be higher in fatigued MS patients, most studies could not find a similar association (Biberacher et al., 2018, Tedeschi et al., 2007, Nourbakhsh et al., 2016, Bakshi et al., 1999). Yet, more recent studies have rather consistently found associations between fatigue and structural damage to specific parietal and frontal regions (Nourbakhsh et al., 2016, Sepulcre et al., 2009, Pellicano et al., 2010). These findings may indicate that atrophy and lesion formation in specific locations are better predictors of the development of fatigue complaints than global structural damage. This description of fatigue based on local disturbances is consistent with the present finding that dFC of specific regions is a better predictor of fatigue than global dynamic connectivity. An interesting direction for future studies, based on this finding, would be the three-way relationship of local structural damage (white matter lesions, gray matter lesions, or atrophy) with dynamic functional connectivity and fatigue in MS patients.

This study is not without limitations. First of all, in this study we analyzed dFC using a sliding-window technique, which has some inherent methodological caveats (Lurie et al., 2020). Measures of dFC are sensitive to methodological and physiological noise, which could have confounded our analysis. To the best of our abilities, we tried to control for these factors by calculating multiple dFC measures, using a tapered sliding window, and implementing a null model for testing for dFC. Another potential confounder is hemispheric asymmetry due to handedness, though this effect may be limited by our bilateral approach in the analysis of regional connectivity (Liu et al., 2009). Overall, future studies should use longer scans with more data points, advanced measures of dFC, and in-depth analysis of different resting-state functional networks. These studies could further clarify the relationship between dFC and fatigue in MS patients using an approach based on network analysis and graph theory. Secondly, as we did not include sFC in the regression model in order to prevent collinearity, we cannot draw any conclusions about the additional relevance of sFC in a combination with dFC and fatigue. Lastly, the patient group was small and rather heterogeneous. For instance, we included patients that recently switched to second-line medication, who likely experienced a more aggressive disease course prior to the study. The change in medication could have influenced dFC or fatigue severity through differences in moderation of acute inflammation and lesion formation. Though our study did not show changes in any clinical measures for the two medication groups from baseline to follow-up, future studies into the relationship between dynamic connectivity and fatigue could try to include a larger patient group with dFC measurements at multiple time points. This will increase statistical power and allow for a more nuanced analysis of the relationship between dynamic connectivity and fatigue in different subpopulations. These studies should also try to address fatigue in the context of comorbidities known to accompany fatigue, such as depression and sleep disorders. Finally, future studies could look at the translatability of the relationship between dynamic connectivity and fatigue to different clinical populations or healthy populations.

## 5 Conclusion

The brain network of MS patients is globally more dynamic than the healthy brain, which does not relate to fatigue in either group. However, lower dFC of specific connections between the basal ganglia and cortical regions within the DMN relates to greater baseline fatigue in MS patients. These findings indicate that dFC may be used as an additional neural correlate of fatigue in MS and highlight the regional specificity of dFC (in this case, of the basal ganglia and DMN) in explaining this symptom. Gaining further understanding of dFC as a neural correlate of fatigue could help in elucidating the mechanisms behind fatigue in MS, which are still poorly captured by conventional MRI measures. Eventually, future studies could explore whether dFC may be used as a potential target for early management of fatigue in the MS population.

## CRediT authorship contribution statement

**Floris B. Tijhuis:** Conceptualization, Formal analysis, Writing - original draft, Visualization. **Tommy A.A. Broeders:** Software, Writing - review & editing. **Fernando A.N. Santos:** Methodology, Writing - review & editing. **Menno M. Schoonheim:** Software, Methodology, Writing - review & editing. **Joep Killestein:** Investigation, Writing - review & editing. **Cyra E. Leurs:** Investigation, Writing - review & editing. **Quinten van Geest:** Software, Methodology, Investigation, Writing - review & editing. **Martijn D. Steenwijk:** Software, Methodology, Writing - review & editing. **Jeroen J.G. Geurts:** Supervision, Writing - review & editing. **Hanneke E. Hulst:** Conceptualization, Writing - review & editing, Project administration, Funding acquisition. **Linda Douw:** Conceptualization, Writing - review & editing, Project administration, Validation.
