# Supplementary material for: Dynamic functional connectivity as a neural correlate of fatigue in multiple sclerosis
Source: Neuroimage Clin. 2021 Jan 4;29:102556. doi: 10.1016/j.nicl.2020.102556 (PMC7815811; doi:10.1016/j.nicl.2020.102556)
Supplement: Supplementary data 1 [file mmc1.pdf]

Supplementary Materials

Table S1. Dynamic functional connectivity obtained from real data and surrogate data.

	Real (*n*=54)	Surrogate (*n*=54)	Test Statistic	*p*
Global dFC-diff	3.32 (0.27)	3.23 (0.25)	Z = -4.938	< 0.001*
Global dFC-cv	0.59 (0.04)	0.58 (0.04)	Z = -4.266	< 0.001*
BC dFC-diff	3.13 (0.33)	3.03 (0.29)	t(53) = 4.994	< 0.001*
BC dFC-cv	0.53 (0.05)	0.52 (0.05)	Z = -3.603	< 0.001*

Data shown here are mean (standard deviation). dFC-diff = dynamic functional connectivity – summed difference method; dFC-cv = dynamic functional connectivity – coefficient of variation; BC = basal ganglia-DMN.

Table S2. Disease characteristics of the two medication groups.

		FIN (*n*=17, *n*=17)	FDMT (*n*=18, *n*=16)	Test Statistic	*p*
Disease duration[a]		10.15 (8.20)	11.75 (5.77)	U = 117.5	0.245
EDSS[b]	T0	3.00 (1.00-6.00)	2.50 (1.50-6.00)	U = 106	0.126
	T1	3.00 (1.50-7.00)[c]	2.75 (1.50-6.00)	U = 103.5	0.361
CIS-20r[a]	T0	71.87 (27.93)[c]	73.07 (24.30)[d]	U = 114	0.444
	T1	76.60 (27.90)[c]	71.53 (30.81)	U = 121	0.809

FIN = fingolimod; FDMT = first-line disease-modifying treatment; EDSS = Expanded Disability Status Scale; T0 = Baseline; T1 = 6-month follow-up

[a] Data shown are mean (standard deviation). [b] Data shown are median (range). [c] *n*=16. [d] *n*=17.

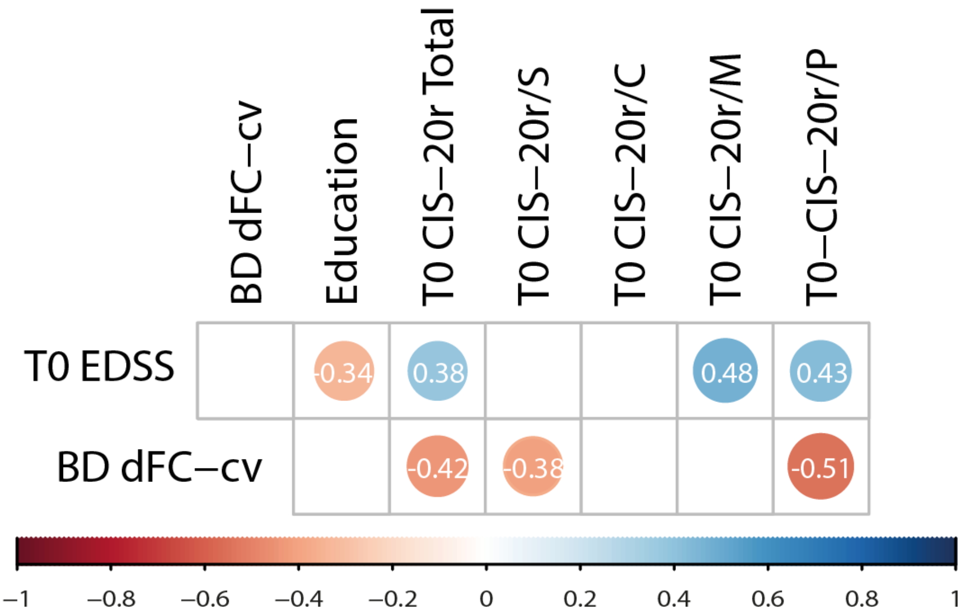

Figure S1. Correlations between predictors in the regression model and fatigue scores in multiple sclerosis patients. The size and color of the circle represents strength and direction of the correlation and the number corresponds to Spearman's rho correlation coefficient. Only the correlations where p<0.05 are shown. T0 = Baseline; BD = basal ganglia-DMN; dFC-cv = dynamic functional connectivity – coefficient of variation; EDSS = Expanded Disability Status Scale; CIS-20r = Revised Checklist of Individual Strength; S = subjective complaints; C = concentration; M = motivation; P = physical activity.
